# Willingness to Participate in Deprescribing Trials: A Survey of Older Adults in Two Countries

**DOI:** 10.1111/jgs.70186

**Published:** 2025-10-23

**Authors:** Sarah E. Vordenberg, Noelia Dulo, Alexander Chaitoff, Kirsten Ingwersen, Kristie Rebecca Weir

**Affiliations:** ^1^ Department of Clinical Pharmacy University of Michigan College of Pharmacy Ann Arbor Michigan USA; ^2^ University of Michigan Center for Bioethics and Social Sciences in Medicine Ann Arbor Michigan USA; ^3^ University of Michigan School of Public Health Ann Arbor Michigan USA; ^4^ Department of Internal Medicine University of Michigan School of Medicine Ann Arbor Michigan USA; ^5^ Sydney Health Literacy Lab, Sydney School of Public, Faculty of Medicine and Health University of Sydney Sydney Australia; ^6^ Leeder Centre for Health Policy, Economics, and Data, Sydney School of Public, Faculty of Medicine and Health University of Sydney Sydney Australia; ^7^ Institute of Primary Health Care (BIHAM) University of Bern Bern Switzerland

**Keywords:** content analysis, decline, deprescribe, preferences, survey

## Abstract

**Background:**

This study investigates the willingness of older adults to participate in a hypothetical deprescribing clinical trial.

**Methods:**

We conducted an online survey of adults aged 65+ years in Australia and the United States. Participants rated their willingness to enroll in a deprescribing trial, responding to the statement, “Research is conducted to assess the safety and effectiveness of stopping medicines. Imagine your doctor made you aware of a research trial aiming to help people stop one or more of their medicines. To what extent would you be willing to enroll in the study?” on a 6‐point Likert scale with “Not at all willing (1)” and “Extremely willing (6)” as the scale anchors. Participants provided a brief free‐text explanation. We dichotomized the outcome variable as willing (scores 4–6) and unwilling (scores 1–3) to enroll and conducted descriptive analyses, chi‐square tests, and univariate and multivariate logistic regression models. Free‐text responses were analyzed using content analysis, with descriptive statistics summarizing themes.

**Results:**

There were 2334 participants in the quantitative analysis and 2237 participants in the content analysis. Most were willing (*n* = 1705, 73%) rather than unwilling (*n* = 629, 27%) to enroll in a deprescribing trial (*p* < 0.001, 95% CI 0.712, 0.748). Over one‐half of participants (*n* = 1252, 56%) expressed the “positive about deprescribing trials” domain, with Australian participants more likely to do so (AU 666 [60%] vs. US 586 [52%], *p* < 0.001). Participants (*n* = 1047, 47%) frequently reported at least one theme of the “concerns and hesitations” domain (*n* = 669, 30%) with US participants more frequently expressing negative views (US 273 [24%] vs. AU 211 [19%], *p* = 0.002) and reporting the “mistrust” theme (US 74 [7%] vs. AU 35 [3%], *p* < 0.001).

**Conclusions:**

Older adults showed a willingness to engage in deprescribing trials, though concerns may affect enrollment. Clear communication of risks and benefits could support recruitment.

**Summary**

- Key points
  ○ Older adults are often underrepresented in deprescribing research trials due to arbitrary age limits, exclusion criteria, and practical burdens of study protocols.
  ○ Quantitative analysis showed that 73% of Australian and American participants reported being willing to enroll in a hypothetical deprescribing trial; however, nearly 47% of participants shared negative perceptions, concerns, or hesitations via free‐text comments.
  ○ Australian participants were more likely to express positive views about enrolling in a deprescribing trial, while American participants more often reported negative views, particularly mistrust.
- Why does this paper matter?
  ○ Our study highlights a willingness of older adults to participate in deprescribing trials yet reveals underlying complexities and hesitations that could impact enrollment. Our findings reinforce the importance of tailoring recruitment strategies to address these reservations through clear, transparent communication and educational efforts.

## 1 Introduction

Deprescribing, the supervised withdrawal of medications by healthcare professionals, is increasingly advocated as a strategy to reduce inappropriate medications [1]. Deprescribing clinical trials are important to establish the safety and effectiveness of medication de‐escalation or stopping. However, older adults with multimorbidity and polypharmacy are often underrepresented in these trials due to arbitrary age limits, exclusion criteria (e.g., cognitive impairment, polypharmacy), and the practical burdens of study protocols that can be challenging for frail individuals [2, 3, 4]. Although guidelines promote inclusion to ensure safety and efficacy data are generalizable [5, 6, 7], these gaps remain.

Despite high stated willingness to deprescribe in surveys [8, 9, 10], deprescribing trials often report substantial refusal rates. In six deprescribing clinical trials, 70%–85% of older adults who were approached declined to participate, though specific reasons were mostly unreported [11, 12, 13, 14, 15, 16]. This discrepancy highlights a gap between stated willingness to deprescribe and actual willingness to enroll in deprescribing research.

To our knowledge, only one paper has explored reasons for patient refusal to participate in deprescribing studies [17]. Understanding patients' perspectives is key for designing engaging recruitment strategies that make trials more inclusive and representative.

## 2 Methods

We conducted a 15‐min survey among adults 65 years and older in Australia and the United States (US). The University of Sydney Human Research Ethics Committee (2024/HE001357) and the University of Michigan Institutional Review Board (HUM00259517) approved the study. We followed the Guideline for RepOrting Vignette Experiments (GROVE) [18].

### 2.1 Study Design and Sample

The study recruited participants between November 2024 and January 2025 from internet panels, comprised of individuals who had opted in to participate in internet‐based surveys, in Australia (Dynata: Shelton, CT) and in the US (Qualtrics Research Panels: Provo, UT). Randomly selected eligible panels were invited to achieve a target of 1200 participants per country, with quotas for age, gender, education, and race and ethnicity in the US. Sampling continued until these quotas were met, using methods like IP verification and digital fingerprinting to prevent duplicate submissions. The survey invitations did not disclose the study's topic to minimize self‐selection bias. Data were collected anonymously using Qualtrics software (Provo, UT), and participants received compensation as per their panel agreement terms.

### 2.2 Survey

We developed a survey to explore older adults' preferences for starting and stopping chronic medication via a hypothetical vignette. Then, they rated their willingness to enroll in a deprescribing trial in response to the statement, *“Research is conducted to assess the safety and effectiveness of stopping medicines. Imagine your doctor made you aware of a research trial aiming to help people stop one or more of their medicines. To what extent would you be willing to enroll in the study?”* on a 6‐point Likert scale with “Not at all willing (1)” and “Extremely willing (6)” as the scale anchors. Participants provided a brief free‐text explanation for their response.

We measured three classes of covariates: (1) health‐related characteristics, (2) attitudes towards healthcare, and (3) demographic characteristics. Health‐related characteristics included self‐reported health [19], the number of prescription medications and non‐prescription medications and/or dietary supplements taken in a typical week, and health literacy, assessed by a single question regarding confidence in filling out medical forms [20, 21]. Attitudes towards healthcare were measured via the 10‐item Wake Forest Trust scale [22], 8‐item Attitudes Towards Uncertainty scale [23], and 6 items related to health promotion from the Health Regulatory Focus Scale [24]. We also collected information about participants' age, country of residence, gender, and highest level of education.

### 2.3 Data Analysis

#### 2.3.1 Quantitative Analysis

We used descriptive statistics to summarize the demographic characteristics, health‐related characteristics, and attitudes towards healthcare of the study sample. For our primary analyses, we dichotomized the outcome variable as willing (scores 4–6, coded as 1 for models) and unwilling (scores 1–3, coded as 0 for models) to enroll in a hypothetical deprescribing trial. We used Chi‐Square Goodness‐of‐Fit testing to test the hypothesis that there was no difference between the proportion of participants willing versus unwilling to participate in a deprescribing trial. We then used univariate regression models to assess the association between each of the independent variables and the outcome. Finally, we used a multivariate logistic regression model to assess the effect of each of these independent variables on the outcome while adjusting for the others. As a secondary analysis, we kept the outcome variable ordinal (scale 1–6) and used multivariable ordinal regression to examine associations between each predictor and the more granular willingness categories. Statistical significance was defined as *p* < 0.05. Analyses were conducted using R (Version 2024.12.1+563).

#### 2.3.2 Content Analysis

We conducted a content analysis of free‐text responses, blending quantitative and qualitative methods to examine the content and frequency of codes [25]. The comments were organized in Excel, version 2502 (MicroSoft Corporation). Codes were developed deductively (informed by relevant literature [17, 26]) and inductively by three investigators (K.R.W., N.D. and S.E.V), who read through the free‐text responses and created initial codes. The final coding framework of 19 codes was tested on 300 randomly selected responses, showing moderate agreement (*κ* = 0.74, 200 responses) initially and high agreement (*κ* > 0.80, 100 responses) after additional training. K.R.W. and N.D. analyzed data from March 2025 to May 2025. Descriptive statistics assessed the frequency of each code, and similar codes were merged. We used two themes, no answer and unclear, to exclude responses that could not be coded. The final analysis framework included 15 themes across four domains. We examined whether content analysis domains and themes differed between countries, using a statistical significance level of *p* < 0.05. We reported the frequency of each theme and domain by participant using Stata SE 18.0 (StataCorp).

## 3 Results

A total of 4464 individuals entered the survey, of which 1970 were excluded due to sampling quotas for their demographic group already being met, and 74 were excluded because they were less than 65 years old. Several participants (*n* = 86) did not respond to the quantitative question about their willingness to enroll in a deprescribing trial. A small number of participants (*n* = 97) did not provide a free‐text response that could be coded. The final analytical sample included 2334 participants in the quantitative analysis and 2237 participants in the content analysis.

Participants (*n* = 2334) reported a median age of 70 and were divided between Australia (*n* = 1148, 49%) and the US (*n* = 1186, 51%) (Table 1). Participants frequently reported that their highest level of education was an Associate's degree, some college, or trade school (*n* = 876, 38%). Participants reported taking a median of 4 medications (IQR 2, 7) and being in good health (*n* = 1038, 45%). Most participants (*n* = 1402, 60%) reported being extremely confident in filling out medical forms.

**TABLE 1:** Older adults' demographic characteristics by willingness to enroll in a deprescribing trial (*n* = 2334).

	Number of participants (%) or median and (interquartile range)
Demographic characteristics	
Age in years	70 (67, 74)
Country	
Australia	1148 (49)
United States	1186 (51)
Gender	
Male	1200 (51)
Female	1129 (48)
Another gender	5 (0)
Education	
High school diploma or less	684 (29)
Associate's degree	876 (38)
Bachelor's degree	499 (21)
Master's degree or higher	275 (12)
Health‐related characteristics	
Total number of medications	4 (2, 7)
Prescription	3 (1, 5)
Non‐prescription	1 (0, 3)
Health status	
Excellent	99 (4)
Very good	572 (25)
Good	1038 (45)
Fair	534 (23)
Poor	91 (4)
Health literacy^a^	
Extremely	1402 (60)
Quite a bit	647 (28)
Somewhat	206 (9)
A little bit	39 (2)
Not at all	40 (2)
Attitudes towards healthcare	
Trust in the doctor^b^	43 (36, 49)
Attitude towards uncertainty^c^	32 (28, 36)
Health promotion^d^	27 (23, 30)
Willingness to enroll in a hypothetical deprescribing trial	
1‐Not at all willing	227 (10)
2	146 (6)
3	256 (11)
4	508 (22)
5	572 (25)
6‐Extremely willing	625 (27)

^a^ Confidence filling out medical forms [20, 21].

^b^ Scores between 5 (low trust) and 50 (high trust).

^c^ Scores between 5 (low) and 40 (high preference towards certainty).

^d^ Scores between 6 (low) and 42 (high preference towards health promotion activities).

### 3.1 Quantitative Analysis

Overall, most participants reported being willing (*n* = 1705, 73%) rather than unwilling (*n* = 629, 27%) to enroll in a deprescribing trial that their doctor made them aware of (*p* < 0.001, 95% CI 0.712, 0.748). In unadjusted analyses, there were associations between all covariates except for age (*p* = 0.600) and self‐reported health status (*p* = 0.200) and willingness to participate in a deprescribing trial (Table 2 and Table S1). Notably, having more than a high school education, having higher health literacy, taking more medications, being female, being from Australia, and having higher ratings on all attitudes towards healthcare scales were associated with a higher likelihood of being willing to participate in a deprescribing trial. These associations, except for those between total number of medications and health literacy with willingness to participate, were present in the adjusted analysis (Table 2). Specifically, after adjusting for all covariates, participants from the US were less likely to report being willing to enroll in a deprescribing trial than Australian participants (OR 0.61, 95% CI 0.50, 0.74). The factors associated with participants' willingness to enroll in a deprescribing trial, holding the value of the covariates constant, included being female as opposed to male (OR 1.35, 95% CI 1.11, 1.64); reporting an Associate's degree, some college, or trade school (OR 1.27, 95% CI 1.01, 1.60), Bachelor's degree (OR 1.45, 95% CI 1.11, 1.92), or Master's degree or higher (OR 1.54, 95% CI 1.10, 2.17) as opposed to a high school diploma or less; higher trust in their doctor (OR 1.01, 95% CI 1.00, 1.03); a preference for more certainty (OR 1.05, 95% CI 1.03, 1.07); and a desire to engage in more health promotion activities (OR 1.03, 95% CI 1.01, 1.05).

**TABLE 2:** Older adults' willingness to enroll in a deprescribing trial based on unadjusted and adjusted logistic regression analysis (unwilling vs. willing to enroll).

	Unadjusted logistic regression		Adjusted logistic regression	
	Odds ratio (95% CI)	*p*	Odds ratio (95% CI)	*p*
Demographic characteristics				
Age	1.01 (0.99, 1.02)	0.500	1.00 (0.98, 1.01)	0.700
Country				
Australia	REF	**< 0.001**	REF	**< 0.001**
United States	0.63 (0.52, 0.76)		0.61 (0.50, 0.74)	
Gender				
Male	REF	**< 0.001**	REF	**0.003**
Female	1.40 (1.16, 1.68)		1.35 (1.11, 1.64)	
Education				
High school diploma or less	REF	**0.014**	REF	**0.018**
Associate's degree	1.30 (1.04, 1.63)		1.27 (1.01, 1.60)	
Bachelor's degree	1.45 (1.12, 1.88)		1.45 (1.11, 1.92)	
Master's degree or higher	1.45 (1.06, 2.01)		1.54 (1.10, 2.17)	
Health‐related characteristics				
Total number of medications	1.10 (1.00, 1.03)	**0.037**	1.01 (1.00, 1.03)	0.067
Health status				
Excellent or very good	1.01 (0.79, 1.28)	0.200	0.88 (0.67, 1.16)	0.130
Good	1.19 (0.95, 1.48)		1.12 (0.88, 1.42)	
Fair or poor	REF		REF	
Health literacy				
Extremely	1.84 (1.40, 2.40)	**< 0.001**	1.41 (1.05, 1.89)	0.068
Quite a bit	1.61 (1.20, 2.16)		1.37 (1.00, 1.87)	
Somewhat or less	REF		REF	
Attitudes towards healthcare				
Trust in the doctor	1.04 (1.02, 1.05)	**< 0.001**	1.01 (1.00, 1.03)	**0.036**
Attitude towards uncertainty	1.07 (1.06, 1.09)	**< 0.001**	1.05 (1.03, 1.07)	**< 0.001**
Health promotion	1.04 (1.02, 1.05)	**< 0.001**	1.03 (1.01, 1.05)	**0.002**

*Note:* Statistically significant at *p* < 0.05 and are reported in bold font.

When leaving the outcome variable ordinal as a secondary analysis, there was substantial variability in the responses, ranging from 26.8% (*n* = 625) reporting being “extremely willing” compared to 9.7% (*n* = 227) who were “not at all willing” to enroll (Table S2). Similar findings were observed when conducting unadjusted and adjusted ordinal regressions compared to the previously reported logistic regression analysis (Table S3).

### 3.2 Content Analysis

Over one‐half of participants (*n* = 1252, 56%) expressed the “positive about deprescribing trials” domain (Table 3). Participants (*n* = 1047, 47%) also frequently reported at least one theme related to the “concerns and hesitations” domain (*n* = 669, 30%) or “negative about deprescribing trials” domain (*n* = 484, 22%). Australian participants more frequently expressed the “positive about deprescribing trials” domain (AU 666 [60%] vs. US 586 [52%], *p* < 0.001). In contrast, US participants more often reported themes within the “negative about deprescribing trials” domain (AU 211 [19%] vs. US 273 [24%], *p* = 0.002), particularly “mistrust” (AU 35 [3%] vs. US 74 [7%], *p* < 0.001).

**TABLE 3:** Prominent domains and themes identified in free‐text responses.

Domain and theme	Example quote	Frequency (%)			*p*
		Overall (*n* = 2237)	Australia (*n* = 1113)	United States (*n* = 1124)	
Positive about deprescribing trials		1252 (56)	666 (60)	586 (52)	**< 0.001**
Preference for deprescribing	*“Been taking medication now for over 20 years. If there was a trial that would have me take less pills, your darn right I would enroll”* *“I think it would help me be reassured that I have made the right decision about the medications I am taking”*	542 (24)	270 (24)	272 (24)	0.974
Helping themselves and others	*“If research can prove that discontinuing medication is safe and effective under specific conditions, it will provide a new treatment option for more patients and reduce their financial and psychological burden”*	436 (19)	235 (21)	201 (18)	0.054
Advancing research and medical knowledge	*“Anything to help research”* *“Without research, medical advancements would not occur”*	217 (10)	134 (12)	83 (7)	**< 0.001**
General interest in participating in a deprescribing trial	*“Happy to try it out and see if I could stop”* *“I'd give it a go”*	200 (9)	105 (9)	95 (8)	0.416
Reduced or minimal risk when deprescribing in a research setting	*“A trial with doctors there with me all the way would ease my mind. I would be happy to get of[f] my pills”* *“I would be interested because it would be a controlled experiment and if my health was at risk, I would be directed to go back to taking the medicine”*	47 (2)	30 (3)	17 (2)	0.051
Concerns and hesitations		669 (30)	320 (29)	349 (31)	0.235
Need more information or to think about it	*“I would need to know a lot of facts before I signed up for any trials”* *“I would want more information about the study and what benefits, if any, before I'd want to participate in it”*	258 (12)	120 (11)	138 (12)	0.268
Conditional willingness	*“It would depend on what medication it is and what it treats. If it was something such as cholesterol medicine, I'd be willing to do it on a trial basis. If it was my allergy medication, that wouldn't be possible since I need it daily”*	234 (10)	117 (11)	117 (10)	0.937
Health risks of being in the study	*“It's all about the risks with a new study and side effects. I would be willing but anxious”* *“I would like to be fully informed of any possible side effects and any significant reactions that I might experience”*	232 (10)	111 (10)	121 (11)	0.539
Participation burden	*“The trial would likely be more inconvenient than I would want to endure”*	115 (5)	62 (6)	53 (5)	0.360
Negative about deprescribing trials		484 (22)	211 (19)	273 (24)	**0.002**
Not interested in participating	*“I do not like to do research trials”*	148 (7)	54 (5)	94 (8)	**0.001**
Feel comfortable with their medicines and don't want to change	*“Right now, the medicines I take are controlling my conditions. I do not want to change what is working”* *“I wouldn't enroll in this study because I am comfortable taking my medications. I would rather be safe than sorry”*	139 (6)	70 (6)	69 (6)	0.883
Not a good candidate for a deprescribing trial	*“I am only on 1 medicine, so not worth the trouble”* *“I think, being 79, that I am too old for many such studies”* *“I don't think I would add anything to the study”*	139 (6)	76 (7)	63 (6)	0.231
Mistrust	*“Don't trust doctors, don't trust ‘researchers’ and don't trust pharmaceutical companies”*	109 (5)	35 (3)	74 (7)	**< 0.001**
Personal and contextual factors		273 (12)	149 (13)	124 (11)	0.089
Trust in their doctor	*“I would continue to take the medication until the doctor said otherwise I would have to think very strongly about stopping medicine”* *“I would trust my Dr to have researched this trial and would only recommend if they thought it was suitable for me”*	141 (6)	81 (7)	60 (5)	0.059
Medication or condition specific considerations	*“My medications are needed for depression high cholesterol and reflux”* *“Would be more than happy to stop taking some of my medications, particularly statins”*	137 (6)	71 (6)	66 (6)	0.617

*Note:* Statistically significant at *p* < 0.05 and are reported in bold font.

Some participants (*n* = 154, 7%) had mixed perceptions of participating, as evidenced by reporting at least one theme “positive about deprescribing trials” domain, plus either the “concerns and hesitations” or “negative about deprescribing trials” domains. Participants (*n* = 273, 12%) less frequently reported the “personal and contextual factors” domain. A full description of the domains and themes is provided in Table S4.

## 4 Discussion

Nearly three‐fourths of participants were willing (scores 4–6) to enroll in a hypothetical deprescribing trial if they were made aware by their doctor, but only one‐quarter were extremely willing (score 6) to enroll. Nearly one‐half of the participants expressed negative perceptions about enrolling in the free‐text responses. Participants' interest in enrolling, driven by concerns about side effects, long‐term harm, or unclear medication necessity, aligns with previous research showing that positive views towards deprescribing are often motivated by similar factors and a preference to follow their doctor's recommendation [27, 28].

Komagamine et al. [29] examined acceptance and refusal of a deprescribing intervention in older hospitalized patients (*n* = 136) in Japan and found no significant associations between refusal and measured characteristics (age, gender, comorbidity, number of medications, or number of potentially inappropriate medications). They inferred personal values and preferences likely played a key role, as they did not explore reasons qualitatively. In contrast, we found that being female and having a university degree were associated with greater willingness to participate, while US participants were significantly less likely to be willing than Australians‐perhaps reflecting healthcare system differences or broader cultural attitudes. Our content analysis supported these findings: Australian participants more often expressed positive views, while US participants more frequently reported negative views, such as mistrust. To our knowledge, this is the first direct comparison of its kind between Australia and the US; further research is needed to better understand the reasons behind these differences.

Our content analysis showed that a substantial proportion of participants voiced concerns, hesitations, and resistance to enrolling in a deprescribing trial, aligning with a US study by Strayer et al. [17], who found that participants (*n* = 1226 patients [545 non‐Veterans and 681 Veterans]) declined enrollment due to feeling overwhelmed by their health, mistrust of research, or hesitancy. While their analysis focused only on primary reasons, our approach included demographic and other variables and analyzed all reasons without restriction. Participants in our study raised concerns about health risks, trial demands, and underlying research motivations, supporting the need for communication that builds trust and addresses concerns in future recruitment strategies. Participants in our study wanted more information about the trial's specific purpose, benefits, risks, and supporting evidence. While framing a trial as “medicines optimization” could help reduce patient hesitation, if the main aim is to stop or reduce medications, this may erode trust and lead patients to refuse the intervention or drop out of the trial. To better understand these patterns, future studies should systematically review enrollment rates in deprescribing trials, identifying differences across countries, settings, and between trials focusing on specific medications versus polypharmacy.

Our study's strengths included a large sample size across two countries with diverse healthcare systems, use of recruitment quotas to increase diversity, and the use of online surveys, which older adults often find enjoyable [30]. Limitations included the hypothetical vignette's potential to not fully represent real‐world decision‐making, particularly since we did not provide detailed information about the trial (such as the type of medication or logistics). Although older adults are a heterogeneous group, our sample may not fully capture the perspectives of those in poorer health or who are unable to complete online surveys. In our study, the median number of medications was four, which is broadly consistent with online survey samples and may be comparable to single‐drug class deprescribing trials. However, it is likely to be lower than polypharmacy trials that typically include participants taking ≥ 5 medications.

Our study highlights a willingness of older adults to participate in deprescribing trials yet reveals underlying complexities and hesitations that could impact enrollment. Future research is needed to explore effective ways to address participants' concerns, including across different disease states and medication types.

## Author Contributions

All authors meet the International Committee of Medical Journal Editors (ICMJE) criteria for authorship. S.E.V. and K.R.W. conceptualized the study and led the drafting of the manuscript. K.R.W. and N.D. conducted the content analysis and K.I. conducted the statistical analysis. S.E.V. contributed to all aspects of the analysis. A.C. provided statistical guidance and contributed to the interpretation of data. All authors critically revised the manuscript for important intellectual content and approved the final version for publication.

## Disclosure

The funders had no role in the design, analysis, and preparation of the article. The contents do not represent the views of the U.S. Department of Veterans Affairs or the United States Government.

## Conflicts of Interest

The authors declare no conflicts of interest.

## Supporting information


**Data S1:** jgs70186‐sup‐0001‐supinfo.pdf.
