# Supplementary material for: Willingness to Participate in Deprescribing Trials: A Survey of Older Adults in Two Countries
Source: J Am Geriatr Soc. 2025 Oct 23;74(2):500–8. doi: 10.1111/jgs.70186 (PMC12911535; doi:10.1111/jgs.70186)
Supplement: Supplementary file 1 — Data S1: jgs70186‐sup‐0001‐supinfo.pdf. [file JGS-74-500-s001.pdf]

Supplemental Materials

Supplementary Table 1. Older adults' demographic characteristics by willingness to enroll in a deprescribing trial

Supplementary Table 2. Older adults' demographic characteristics by willingness to enroll in a deprescribing trial

Supplementary Table 3. Older adults' willingness to enroll in a deprescribing trial based on univariate and multivariate ordinal regression analysis

Supplementary Table 4. Description of themes used in the content analysis

Supplementary Table 1. Older adults' demographic characteristics by willingness to enroll in a deprescribing trial

	Number of Participants (%) or Median (Interquartile Range)	
	**Not willing (Score 1-3)**	**Willing (Score 4-6)**
**Demographic characteristics**		
Age	70 (67, 74)	70 (67, 74)
Country		
Australia	257 (41)	891 (52)
United States	372 (59)	814 (48)
Gender[a]		
Male	361 (58)	839 (49)
Female	266 (42)	863 (51)
Education		
High school diploma or less	215 (34)	469 (28)
Associate's degree	228 (36)	648 (38)
Bachelor's degree	120 (19)	379 (22)
Master's degree or higher	66 (10)	209 (12)
**Health-related characteristics**		
Total number of medications	4 (2, 6)	4 (2, 8)
Health Status		
Excellent/very good	190 (30)	481 (28)
Good	261 (41)	777 (46)
Fair/poor	178 (28)	447 (26)
Health Literacy		
Extremely	346 (55)	1,056 (62)
Quite a bit	176 (28)	471 (28)
Somewhat or less	107 (17)	178 (10)
**Attitudes towards healthcare**		
Trust in the doctor[b]	40 (33, 48)	43 (37, 49)

Attitude towards uncertainty[c]	31 (26, 35)	33 (29, 37)
Health promotion[d]	26 (21, 29)	27 (23, 30)

[a] Participants who identified as another gender or preferred not to report their gender were not included in this analysis due to the small number (n=5)
[b] Scores between 5 (low trust) and 50 (high trust)
[c] Scores between 5 (low) and 40 (high preference towards certainty)
[d] Scores between 6 (low) and 42 (high preference towards health promotion activities)

Supplementary Table 2. Older adults' demographic characteristics by willingness to enroll in a deprescribing trial

	Number of Participants (%) or Median (Interquartile Range)					
	Not at all willing					Extremely willing
	1	2	3	4	5	6
**Demographic characteristics**						
Age	69 (67, 74)	70 (67, 74)	70 (67, 74)	69 (67, 74)	70 (67, 75)	70 (67, 74)
Country						
Australia	75 (33)	55 (38)	127 (50)	237 (47)	309 (54)	345 (55)
United States	152 (67)	91 (62)	129 (50)	271 (53)	263 (46)	280 (45)
Gender						
Male	140 (62)	79 (54)	142 (56)	267 (53)	267 (47)	305 (49)
Female	86 (38)	67 (46)	113 (44)	240 (47)	304 (53)	319 (51)
Education						
High school diploma or less	83 (37)	43 (29)	89 (35)	143 (28)	154 (27)	172 (28)
Associate's degree	84 (37)	59 (40)	85 (33)	190 (37)	206 (36)	252 (40)
Bachelor's degree	40 (18)	25 (17)	55 (21)	113 (22)	144 (25)	122 (20)
Master's degree or higher	20 (8.8)	19 (13)	27 (11)	62 (12)	68 (12)	79 (13)
**Health-related characteristics**						
Total number of medications	4 (2, 6)	4 (2, 6)	4 (2, 6)	5 (2, 7)	4 (2, 7)	5 (2, 8)
Health Status						

Excellent/very good	89 (39)	32 (22)	69 (27)	130 (26)	145 (25)	206 (33)
Good	82 (36)	71 (49)	108 (42)	243 (48)	278 (49)	256 (41)
Fair/poor	56 (25)	43 (29)	79 (31)	135 (27)	149 (26)	163 (26)
Health Literacy						
Extremely	143 (63)	73 (50)	130 (51)	266 (52)	322 (56)	468 (75)
Quite a bit	52 (23)	50 (34)	74 (29)	177 (35)	196 (34)	98 (16)
Somewhat or less	32 (14)	23 (16)	52 (20)	65 (13)	54 (9.4)	59 (9.4)
**Attitudes towards healthcare**						
Trust in the doctor	42 (32, 48)	40 (31, 46)	40 (34, 49)	41 (35, 48)	42 (37, 49)	46 (39, 50)
Attitude towards uncertainty	31 (25, 35)	30 (25, 33)	31 (27, 35)	31 (27, 35)	32 (29, 36)	36 (31, 39)
Health promotion	27 (22, 31)	26 (21, 29)	25 (21, 29)	26 (22, 30)	27 (23, 30)	28 (24, 32)

Supplementary Table 3. Older adults' willingness to enroll in a deprescribing trial based on univariate and multivariate ordinal regression analysis

	**Unadjusted Ordinal Regression**		**Adjusted Ordinal Regression**	
	**Odds Ratio (95% C.I.)**	**p-value**	**Odds Ratio (95% C.I.)**	**p-value**
**Demographic characteristics**				
Age	1.01 (0.99, 1.02)	0.300	1.00 (0.99, 1.01)	>0.900
Country				
Australia	REF	**<0.001**	REF	**<0.001**
United States	0.64 (0.56, 0.74)		0.62 (0.54, 0.72)	
Gender				
Male	REF	**<0.001**	REF	**0.001**
Female	1.31 (1.13, 1.51)		1.28 (1.10, 1.49)	
Education				
High school diploma or less	REF	0.061	REF	0.120
Associate's degree	1.23 (1.03, 1.48)		1.18 (0.99, 1.42)	
Bachelor's degree	1.22 (1.00, 1.49)		1.19 (0.97, 1.47)	
Master's degree or higher	1.31 (1.02, 1.68)		1.33 (1.02, 1.72)	
**Health-related characteristics**				
Total number of medications	1.02 (1.01, 1.03)	**0.003**	1.01 (1.00, 1.02)	**0.012**
Health Status				
Excellent or very good	1.08 (0.89, 1.32)	0.700	0.96 (0.77, 1.18)	0.800

Good	1.05 (0.88, 1.25)		1.01 (0.84, 1.21)	
Fair or poor	REF		REF	
Health Literacy				
Extremely	1.86 (1.48, 2.33)		1.32 (1.04, 1.68)	
Quite a bit	1.17 (0.92, 1.49)	**<0.001**	0.95 (0.74, 1.22)	**<0.001**
Somewhat or less	REF		REF	
**Attitudes towards healthcare**				
Trust in the doctor	1.04 (1.03, 1.05)	**<0.001**	1.01 (1.00, 1.02)	0.022
Attitude towards uncertainty	1.10 (1.08, 1.11)	**<0.001**	1.07 (1.06, 1.09)	**<0.001**
Health promotion	1.05 (1.03, 1.06)	**<0.001**	1.03 (1.01, 1.04)	**<0.001**

Supplementary Table 4. Description of themes used in the content analysis

Theme	Description
**Positive about deprescribing trials**	
Preference for deprescribing	Preference to reduce or stop medications due to concerns about side effects, long-term harm, the burden of medication management, and the belief that some medications are unnecessary or ineffective. Some considered this an opportunity to confirm whether their medications are needed and to feel reassured about their current treatment decisions.
Helping themselves and others	Research could benefit their own health or the health of others. Volunteering to do research can have personal rewards and create a sense of generosity.
Advancing research and medical knowledge	Contributing to research that could lead to cures, better treatments, and scientific advancements for future generations. They valued helping to expand medical knowledge and improve healthcare outcomes.
General interest in participating in a deprescribing trial	General interest to be part of a deprescribing trial.
Reduced or minimal risk when deprescribing in a research setting	Perceived minimal risk or reduced harm in participating in a deprescribing trial, considering the safety measures and oversight involved in a clinical trial as reassuring.
**Concerns and hesitations**	
Need more information or to think about it	Need more information about the trial before deciding, including details about the trial's purpose, potential benefits and harms, and how it would affect their health. They mentioned feeling unsure, needing time to think, to weigh the pros and cons, to understand the specific medications involved, and see supporting evidence before committing to participate.
Conditional willingness	Participation in the trial was seen as conditional on various factors such as the specific medication and condition involved, the time and duration of the study, regular monitoring of health risks, and the convenience or compensation offered. Participants also weighed whether the medication was still needed, the stage of the trial, and the potential outcomes and who was running the research, expressing more trust in university or hospital-led studies than those by pharmaceutical companies.
Health risks of being in the study	Concerns about unspecified health risks and the potential dangers of participating, including risks of stopping or starting medications, long-term health effects, and uncertainties about which medication to stop. Participants also mentioned concern about new health problems emerging or the return of a condition or symptoms if a medication were stopped.

Participation burden	Concerns about the effort, logistics, cost and emotional burden of participating in a deprescribing trial, including time and travel demands, inconvenience, and costs.
**Negative about deprescribing trials**	
Not interested in participating	A lack of interest in participating in the trial, feeling it was not relevant to them, unnecessary, or not worthwhile.
Feel comfortable with their medications and don't want to change	Comfort with their current medications and a reluctance to change what was working. They saw their medications as necessary and beneficial for managing their conditions, maintaining their health, and supporting their overall wellbeing.
Not a good candidate for a deprescribing trial	Participants felt they were not suitable for a deprescribing clinical trial, for reasons like they were not taking any medications, having chronic conditions, or they had concerns about their age, life expectancy, mental health, or overall health status.
Mistrust	Mistrust of clinical trials, healthcare providers, researchers, and the pharmaceutical industry. Many did not want to be treated as test subjects or "guinea pigs" and doubted the value or motivations of research involving medications.
**Personal and contextual factors**	
Trust in their doctor	Trust in their doctor’s expertise and guidance, often deferring to their doctor’s judgment on whether to participate in a deprescribing trial. Many said they would discuss joining the trial with their doctor first and would consider enrolling if their doctor recommended it. Others expressed trust in their doctor as a reason to **not** stop any of their current medications.
Medication or condition specific considerations	Mentioned specific medications or conditions like diabetes, cancer, depression, when discussing whether they would stop medications. Their decisions often hinged on how important they felt the medication was for managing that condition or whether they believed it was causing side effects.
